# Letter to the editor (March 22, 2026) concerning the paper “Psychosocial working conditions and mental well-being of remote and stationary employees: a longitudinal study”

**DOI:** 10.13075/ijomeh.1896.02862

**Published:** 2026

**Authors:** Tian Ruan

**Affiliations:** Yunnan Medical Health College, School of Basic Medicine and Pharmacy, Kunming, China

**Keywords:** occupational health, working hours, remote work, artificial intelligence, mental well-being, AI efficiency paradox

Dear Editor,

I read with great interest the longitudinal study by Żołnierczyk-Zreda et al. [1] recently published in “International Journal of Occupational Medicine and Environmental Health,” which demonstrated that remote workers exhibit better mental well-being over time compared to stationary employees. Their finding that favorable psychosocial working conditions – including greater autonomy and flexibility – contribute to improved psychological outcomes is both timely and significant. However, I wish to draw attention to an emerging and potentially countervailing occupational health challenge that may complicate this optimistic picture: the artificial intelligence (AI) efficiency paradox.

Artificial intelligence has been widely heralded as a transformative force for workplace efficiency, with the implicit promise of reducing workloads and working hours. Yet emerging evidence reveals a counterintuitive phenomenon: workers whose jobs have higher AI relevance tend to work longer, not shorter, hours. Increased coupling of employees with AI systems has been shown to reshape work-related interactions and contribute to loneliness and impaired well-being, including insomnia, across four studies encompassing 794 employees [2]. It should be noted that this strand of evidence remains largely cross-sectional and draws on organizational and behavioural science literatures; causal inference and generalizability to occupational-health populations require further prospective study. Nationally representative U.S. survey data indicate that generative AI has been adopted at work faster than the personal computer, yet the time savings reported by workers amount to only about 1.4% of total work hours, with between 1% and 7% of all work hours currently assisted by generative AI [3]. Furthermore, despite substantial AI investment, aggregate productivity gains remain modest (total factor productivity increase ≤0.66% over 10 years); such modest gains may stem from multiple factors – including uneven implementation, high deployment costs, and lagged productivity effects – and do not, by themselves, demonstrate that task-level efficiency gains fail to translate into reduced working time [4].

In proposing the following mechanisms, I acknowledge that several of them resonate with observations already reported in the organizational and practitioner literature; the original contribution of this letter lies less in introducing novel mechanisms than in translating these emerging organizational observations into an occupational-health research agenda concerning remote work, working time, and associated psychosocial risks. Four interconnected mechanisms underlying this paradox are identified.

First, the task expansion effect: by reducing the friction of individual tasks, AI enables workers to take on responsibilities previously outside their scope, resulting in workload inflation rather than time savings; indeed, generative AI has been demonstrated to reduce the time required for professional writing tasks by 40% while raising output quality by 18% [4]. Second, the boundary blur effect: the conversational, low-barrier nature of AI interactions facilitates work initiation during breaks, commutes, and off-hours, progressively eroding the boundary between work and personal time. Notably, Janc et al. [5] demonstrated in IJOMEH that remote workers already exhibit altered sleep component distributions, and AI-driven boundary erosion may further exacerbate such disruptions. Third, rework overhead: AI-generated outputs frequently require verification and correction, and the ongoing need to master and monitor AI systems generates an additional, often unrecognised workload – job insecurity in human–AI collaboration has been linked to technology-learning anxiety that adversely affects well-being and psychological health [6]. Fourth, the ambient work pattern: AI fosters continuous, low-grade engagement with work tasks, leading to cognitive density that intensifies rather than alleviates the workday [2].

The findings of Żołnierczyk-Zreda et al. [1] raise an important question: could the mental health benefits of remote and flexible work arrangements be partially offset by AI-driven working hour extension? Kim et al. [7] have shown in this Journal that working time mismatch – the discrepancy between desired and actual working hours – increases the risk of depression regardless of the actual hours worked. The AI efficiency paradox may systematically generate such mismatches, as workers find themselves working longer than intended without conscious awareness. This echoes the classical Jevons Paradox in resource economics: when technology increases the efficiency of resource use, consumption of that resource may paradoxically increase–a dynamic now being documented for digital and AI technologies in the labour market [8].

The occupational health implications are substantial. Prolonged working hours are an established risk factor for occupational illness: in a predictive analysis of public health inspectors, extended work hours (>10 h/day) were associated with approx. 40% higher burnout risk [9], and frequent AI use has been linked to elevated burnout among physicians (adjusted OR = 1.20) [10]. At the same time, I wish to acknowledge that the implications of increased work involvement are not uniformly negative. It is important to distinguish between compulsive work behavior (workaholism), which is typically associated with adverse health outcomes, and work engagement, which can represent a positive and energizing form of involvement in work [11]. In this respect, AI use has also been linked to heightened work engagement [12].

Moreover, the empirical evidence on AI and worker well-being remains mixed: longitudinal data from Germany found no significant negative effects of AI exposure on workers' well-being and even suggested improvements in self-reported health status and health satisfaction [13]. The effects of AI are therefore likely to be complex and contingent on contextual factors, and the AI efficiency paradox advanced here should be regarded as a hypothesis warranting prospective testing rather than an established conclusion. If AI adoption systematically drives longer working hours among knowledge workers, it may paradoxically worsen the very occupational health outcomes that flexible work arrangements – as demonstrated by Żołnierczyk-Zreda et al. – were expected to improve. Indeed, a recent editorial in this field observed that algorithmic and AI-driven management systems intensify psychosocial risks, with European Agency for Safety and Health at Work (EU-OSHA) data showing that each one-unit increase in such exposure is associated with a 21% rise in psychosocial risks [14]. I urge researchers in occupational medicine to:

– conduct longitudinal studies – following the commendable methodological approach of Żołnierczyk-Zreda et al. – examining the dose-response relationship between AI exposure and working hours,
– investigate the mediating role of organizational AI usage policies,
– develop evidence-based guidelines for sustainable AI integration in the workplace.

Recognizing the AI efficiency paradox is the first step toward designing interventions that harness AI's productivity benefits without sacrificing worker well-being.

## AI USE

AI-assisted tools were used for language polishing and literature searching during the preparation of this manuscript. The author takes full responsibility for the content.
